# Addressing Motion Blurs in Brain MRI Scans Using Conditional Adversarial Networks and Simulated Curvilinear Motions

**DOI:** 10.3390/jimaging8040084

**Published:** 2022-03-23

**Authors:** Shangjin Li, Yijun Zhao

**Affiliations:** Department of Computer and Information Sciences, Fordham University, New York, NY 10023, USA; sli339@fordham.edu

**Keywords:** MRI, motion blur, deep learning, generative adversarial network (GAN)

## Abstract

In-scanner head motion often leads to degradation in MRI scans and is a major source of error in diagnosing brain abnormalities. Researchers have explored various approaches, including blind and nonblind deconvolutions, to correct the motion artifacts in MRI scans. Inspired by the recent success of deep learning models in medical image analysis, we investigate the efficacy of employing generative adversarial networks (GANs) to address motion blurs in brain MRI scans. We cast the problem as a blind deconvolution task where a neural network is trained to guess a blurring kernel that produced the observed corruption. Specifically, our study explores a new approach under the sparse coding paradigm where every ground truth corrupting kernel is assumed to be a “combination” of a relatively small universe of “basis” kernels. This assumption is based on the intuition that, on small distance scales, patients’ moves follow simple curves and that complex motions can be obtained by combining a number of simple ones. We show that, with a suitably dense basis, a neural network can effectively guess the degrading kernel and reverse some of the damage in the motion-affected real-world scans. To this end, we generated 10,000 continuous and curvilinear kernels in random positions and directions that are likely to uniformly populate the space of corrupting kernels in real-world scans. We further generated a large dataset of 225,000 pairs of sharp and blurred MR images to facilitate training effective deep learning models. Our experimental results demonstrate the viability of the proposed approach evaluated using synthetic and real-world MRI scans. Our study further suggests there is merit in exploring separate models for the sagittal, axial, and coronal planes.

## 1. Introduction

Brain magnetic resonance imaging (MRI) is one of the most important imaging modalities in detecting structural abnormalities of the brain. Nevertheless, it is very sensitive to subject motion and in-scanner head motion is a fundamental source of error in brain MRI due to the procedure’s intrinsically slow and sequential process. Specifically, the raw MRI signals are first encoded in k-space [1] and then converted into the human-recognizable MRI scan using an inverse Fourier transform. Any movement during this process will disrupt the encoded signals and result in blurring and ghosting [2,3], causing misinterpretation and reduced reliability in detecting clinically relevant abnormalities [4].

In the past few years, deep learning [5] (DL) has attracted a great amount of interest due to its remarkable progress in computer vision. In medical image analysis, deep neural networks have been extensively applied to various imaging modalities, including X-rays [6,7], B-scans [8,9], and MRIs [10,11], to help provide greater diagnostic and treatment capabilities. Unsurprisingly, many efforts have been made to address MRI motion artifacts using deep learning-based approaches. We provide a brief survey of these related studies in Section 2.

In this study, we investigate the efficacy of employing a generative adversarial network (GAN) approach to address motion blurs in brain MRI scans. Since GANs are generative models trained to generate realistic synthetic images based on the learned distribution of the ground truth images, our models can improve to a limited extent additional motion artifacts (e.g., rings and salt-and-pepper noise) associated with motions besides blurs. Thus, we denote our model MC-GAN (MC for Motion-artifact Correction) and evaluate the overall image quality improvement achieved by applying MC-GAN to degraded MR Images. Methodology-wise, we cast the problem as a blind deconvolution task where a neural network is trained to guess a blurring kernel that produced the observed corruption. Typically, motion blurs are modeled by a convolution kernel *K*. Formally,

(1) $$I_{B}=I_{S}⊙K+N$$

where $I_{B}$, $I_{S}$, and *N* denote blurred image, ground-truth sharp image, and random noise, respectively. ⊙ denotes the convolution operation. In the blind deconvolution problem one attempts to estimate $I_{S}$ without knowing *K*. In general, such problems are severely under-determined, making finding the corrupting kernel largely intractable. However, in a narrow image domain such as brain MRI scans, existing research has shown that a neural network can learn the properties of the domain sufficiently well to infer a region in the kernel space that hosts the damaging kernel [12,13,14].

Inspired by these findings, our study explores a new blind deconvolution approach under the sparse coding paradigm [15] where every ground truth corrupting kernel is assumed to be a “combination” of a relatively small universe of “basis” kernels. This assumption is based on the intuition that, on small distance scales, patients’ moves follow simple curves and that complex motions can be obtained by combining a number of simple ones. Thus, in our approach, we generated a family of 10,000 small curvilinear kernels with random positions and directions to uniformly populate the space of kernels that corrupt real-world scans. A neural network *N* was trained to reconstruct images corrupted by these “basis” kernels. Consequently, the output image reconstructed by *N* could be thought of as accompanied by an implicit kernel that is a function of the “basis” kernels. We interpret training *N* as learning the basis kernels and applying *N* to unseen images as computing a non-linear combination of the basis that the network deems the most likely cause of the degradation. On the conceptual level, we believe that with a suitably dense basis, a neural network will effectively guess the degrading kernel and reverse some of the damage in the motion-affected real-world scans. Our study explores these concepts, and we are unaware of similar reports in the literature. Based on the promise shown by our experiments, the framework presented here merits further investigation.

Data scarcity is another issue we addressed in training our models. Typically, deep learning approaches require big data to avoid overfitting [16]. To this end, we adopted the data augmentation technique introduced in [11] and generated 225,000 synthetic artifact-free (sharp) MR images. Leveraging our large collection of random convolutional kernels described above, we further generated a blurred counterpart for each of the 225,000 images. Consequently, our deep learners were trained to perform blind deconvolution on pairs of corrupted and sharp images, which served as the model input and ground truth, respectively. We illustrate our kernel and synthetic data generation process in Section 4.2.2.

Lastly, this study further evaluates customized models built exclusively for individual MRI planes (i.e., sagittal, axial, and coronal) compared to an omnibus model accommodating input from all three directions. We believe this is an area under-studied in the existing literature, possibly due to the limited availability of labeled data which prevents further partition from building effective sub-models. Capitalizing on our data augmentation techniques, we mitigated the data scarcity issue and exploited the structure similarity in the input data. Indeed, our experimental results indicate that the customized models consistently outperform the general model in all plane directions.

Our study leverages two open-access real-world datasets. First, we generate our synthetic data based on the high-quality MRI scans provided by the open-access OASIS platform [17]. Second, we evaluate the efficacy of our models using held-out synthetic data and motion-affected real-world MRI scans from the ABIDE study [18]. Both quantitative and qualitative model assessments are presented in Section 5.

## 2. Related Work

The existing image blur correction algorithms can be classified into two categories: non-blind deblurring and blind deblurring. The non-blind approaches assume the blurring kernel *K* in Equation (1) is known. Thus, a deblur algorithm performs the deconvolution operation to recover the sharp image $I_{S}$ by treating the randomness of the noise term *N*. Classical algorithms in this domain include Lucy–Richardson deconvolution, an iterative procedure for recovering an underlying image that has been blurred by a known point spread function [19], and Wiener filter-based algorithms [20,21]. In the medical domain, non-blind deconvolution has been applied successfully to remove the noise and blur in the CT image [22], MRI super-resolution [23], and deblurring X-Ray Digital Image [24].

Blind deconvolution, on the other hand, is the recovery of a sharp version of a blurred image when the blur kernel *K* is unknown. It is the most common scenario in real-world applications. Many efforts have been devoted to discovering effective blind deblurring algorithms, which estimate the sharp image ($I_{S}$) and the blur function (*K*) simultaneously. In some early work, Fergus et al. introduced a method to remove the effects of camera shake from seriously blurred images [25]. Xu et al. presented a new framework for both uniform and non-uniform motion deblurring, leveraging an unnatural $L_{0}$ sparse representation to benefit kernel estimation and large-scale optimization [26]. Babacan et al. provided a systematic formulation of blind deconvolution using general sparse image priors [27].

In the past few years, deep learning-based algorithms have delivered promising results in improving the quality of medical images. Sun et al. proposed an effective CNN for estimating motion kernels from local patches [28]. Motion blur is removed by a non-uniform deblurring model using patch-level image prior. Noroozi et al. introduced DeblurNet [29], a novel CNN architecture designed to restore blurry images under challenging conditions, such as occlusions, motion parallax and camera rotations. Gong et al. proposed a flexible and efficient deep learning-based method for estimating and removing the heterogeneous pixel-wise motion blurs. Their model directly estimates the motion flow from the blurred image through a fully-convolutional deep neural network (FCN) and recovers the unblurred image from the estimated motion flow [30].

Although deep learning approaches have been effective in many computer vision tasks, sufficient training data are essential in the success of these models. However, access to medical image data is often limited. To this end, researchers have explored various data augmentation techniques to enhance the size and quality of training datasets [16]. The choice of techniques is highly dependent on the task under investigation. For instance, Eaten-Rosen et al. proposed a novel approach to generate new medical images based on the linear combination of training data [31] in their image segmentation task. He et al. applied flipping and rotation to increase the robustness in abnormality detection in musculoskeletal radiographs [6]. Data augmentation is also utilized to simulate artifacts for the task of correcting degraded medical images. For example, Duffy et al. simulated motion artifacts on MR images to produce synthetic motion-affected data to correct motion artifacts in structural MRI images [32]. Specifically, they simulated the translational motions as multiplications in *k*-space by random linear phase shifts. Zhao et al. simulated “ringing” artifacts via controlled perturbations in the *k*-space representations of MRI scans in their effort to remove the “rings” induced by head motions [11].

Our study likewise employed data augmentation techniques to facilitate training effective deep networks. For the motion-free images, we resorted to the sharp image generation technique introduced in [11]. For the motion-affected images, we augmented the sharp images by applying random curvilinear kernels produced using a probabilistic approach (Section 4.2.2). Our method is in contrast to some existing approaches that simulated the motion artifacts via *k*-space manipulations. We believe that our method is feasible in narrow image domains (e.g., brain MRI scans), and our experimental results endorse this view.

## 3. Methods

In this section, we briefly introduce the generative adversarial network (GAN) framework and illustrate the structure of our MC-GAN model.

### 3.1. Generative Adversarial Network

**Figure 1:**
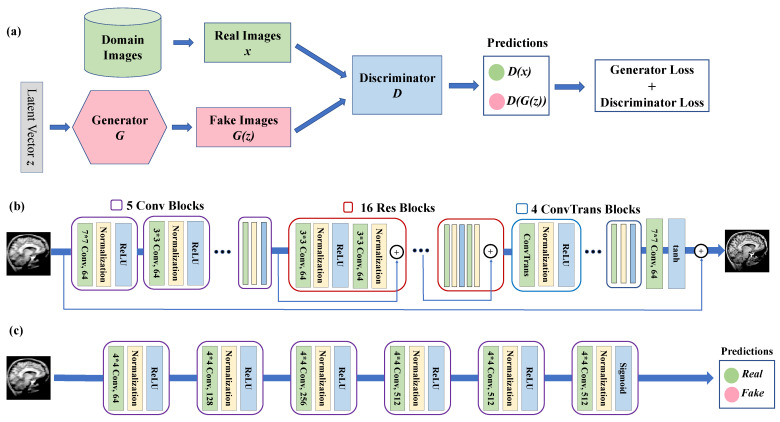
Architecture of MC-GAN Model. (**a**) Overall GAN structure. (**b**) MC-GAN generator. (**c**) MC-GAN discriminator.

A generative adversarial network (GAN) is a class of machine learning approaches introduced by Ian Goodfellow et al. [33]. The core idea of a GAN is to frame a supervised learning problem using two sub-models: a generator (*G*) that generates new instances in the study domain, and a discriminator (*D*) that tries to classify instances as either real (from the domain) or fake (generated). The goal of the generator is to fool the discriminator by generating realistic samples. The two models are trained simultaneously as adversaries in a zero-sum game until the discriminator model is fooled about 50% of the time, which implies the generator is generating realistic examples. Figure 1a illustrates the above adversarial game. Formally, the loss function of a GAN model can be formulated as:

(2) $$\min_{G}\max_{D}\;E_{x}\left[\log\left(D\left(x\right)\right)\right]+E_{z}\left[\log\left(1-D\left(G\left(z\right)\right)\right)\right]$$

where E denotes expectation. *x* is a random variable representing observed real-world images in the study domain. G(z) denotes the generated images by *G* using latent random variable *z*. D(x) and D(G(z)) are *D*’s classification probability of the real and simulated images, respectively. Consequently, the overarching goal of *D* is to maximize the total loss defined in Equation (2), while the goal of *G* is to minimize the second term.

### 3.2. Our Approach

Our MC-GAN was inspired by the successful DeblurGAN model [34], in which the authors presented a conditional GAN (cGAN) [35] approach to recover a sharp image ($I_{S}$) given a single blurred image ($I_{B}$). Unlike traditional GANs, cGAN models provide the generator with additional information to control the scope of the generated images. In the DeblurGAN architecture, the generator learns a mapping from the original image *x* and the latent vector *z* to the output image *y*, i.e., G:x,z→y. Thus, it is natural to employ the ResNet [36] structure where the neural networks strive to learn the difference between the original and target images. Our study followed a similar design but experimentally adjusted the model structure and loss function to overcome issues such as overfitting and model convergence.

### 3.3. MC-GAN Generator

Figure 1b illustrates the structure of MC-GAN’s generator component, which consists of five convolutional blocks to encode and extract spatial features of the input image. Each block contains a convolutional layer, followed by batch normalization and ReLU activation. All kernels are of size 3 × 3 except for the first one, which is 7 × 7.

A total of 16 residual blocks follow the four convolutional blocks. Each residual block contains two 3 × 3 convolutional layers, each followed by a normalization layer and ReLU activation. The architecture ends with four transposed convolutional blocks upscaling the images to the original size. The output after the tanh activation function is in the residual form and, thus, the generator’s output is the original image plus the residual.

### 3.4. MC-GAN Discriminator

The discriminator is a classification model whose input is an image and the output is a probability score indicating if the image is from a real-world domain or generated. We apply the threshold of 0.5 to classify real or fake images.

Figure 1c illustrates the CNN structure of the discriminator component. It consists of seven convolutional blocks. Each block consists of a convolutional layer followed by a normalization layer and an activation function. All kernels are of size 4 × 4 and the number of filters increases from 64 to 512. All blocks use ReLU as the activation function except for the last block, which employs the sigmoid function to produce a probability score for the classification task. As illustrated in Equation (2), the discriminator aims to maximize the total loss, which is equivalent to maximizing the difference between D(x) and D(G(z)).

### 3.5. Loss Function

In the DeblurGan [34] model, the authors formulated the loss function as a combination of adversarial loss from the cGAN model and a perceptual content loss [37] defined as the $L_{2}$ distance between the generated and target image CNN feature maps. The authors further pointed out that DeblurGAN trained without perceptual loss or with simple MSE loss on pixels did not converge to a meaningful state. We believe this is related to the limitation in training GANs, that is, seeking the Nash equilibrium can be very unstable and algorithms may fail to converge [38]. A common technique to encourage model convergence is to augment the adversarial loss with additional loss(es), such as contextual loss [34], or $L_{1}$ loss [32]. In our experiments, we found it was necessary to include additional pixel-wise mean squared error (MSE) in the loss function for our model to converge properly. As a result, following the same notation as in [34], we defined the loss function for our MC-GAN model as a combination of three components:

(3) $$L=L_{GAN}+\lambda L_{X}\;+\beta L_{MSE}$$

where $L_{GAN}$ is the adversarial loss defined in Equation (2), $L_{X}$ is the content perceptual perceptual loss, and $L_{MSE}$ is the pixel-wise content loss. λ and β are trade-off parameters for the three loss components. In our study, they were experimentally set to 100 and 50, respectively.

## 4. Data and Preprocessing

### 4.1. Real-World Datasets

We based our study on two real-world datasets. The first one was the OASIS-1 dataset provided by the Open Access Series of Imaging Studies (OASIS) platform [17]. OASIS-1 contains 436 T1-weighted MRI scans of 416 subjects (Age: 52.7 ± 25.1; Female: 61.5%); 20 subjects had two MRI sessions. All scans were selected through a per-slice screening process along each principal axis to ensure their quality. Each slice is a pixel image of size 256 × 256. Of these, 375 scans from 355 subjects were used to generate our training data, and the remaining 61 scans from 61 subjects were held out for model testing.

Our models were further applied to 55 motion-affected T1-weighted MRI scans selected from the ABIDE study [18]. Of these, we extracted the middle 100 slices along each of the three anatomical planes of each scan, resulting in a test set of 16,500 images. The ABIDE scans were selected from a larger dataset that had been visually evaluated as low quality in a previous study [39]. The size of these test images are the same as the training data (i.e., 256 × 256).

All experiments were performed in accordance with the relevant guidelines and regulations of OASIS-1 and ABIDE studies.

### 4.2. Synthetic Datasets

We address the data scarcity issue in training deep learning models by generating synthetic brain MRI images. To this end, we first generated sharp (i.e., artifact-free) images based on the high-quality OASIS dataset. We then simulated motion blurs on the sharp images using random convolutional kernels.

#### 4.2.1. Generating Synthetic Artifact-Free Data

We generated artifact-free images using the techniques introduced in [11] by modeling the inter-subject brain morphological variability. Specifically, localized deformations were generated on a given sharp image using radial stretches within a randomly selected circular region. The stretches were performed with a smoothly changing ratio to ensure no discontinuity between the modified and unaffected regions. Following the same notation as in [11], the stretching ratio changes according to the following formula:

(4) $$IMG_{new}\left(P\right)=IMG_{old}\left(C+u^{\left(1+\epsilon \right)}\left(P-C\right)\right)$$

where $IMG_{new}\left(P\right)$ is the new pixel intensity at a given point *P* in a circle with the center *C* and radius *R*, and u=distance(P,C)/R. The parameter ϵ was experimentally set to 0.2, and a sample sequence of morphed images can be viewed in succession as shown in this animation [40].

In our study, 50 slices were randomly sampled along each of the sagittal, axial, and coronal directions from each of the 375 scans, resulting in a total of 56,525 images. For each of these images, multiple local spatial distortions were applied to simulate natural inter-subject variability in brain morphology. A total of 225,000 artifact-free images were generated to serve as the ground truth in our training data.

#### 4.2.2. Generate Synthetic Artifact-Affected Data

To model real-world motion kernels, we focused on kernel shapes of short continuous random curves that could result from in-scanner head motion, including rotation, shaking, or nodding. To this end, we generated continuous, non-intersecting random walks of a prescribed length *k*, which started at a random location on a 16 × 16 grid. To achieve the effect of continuity, we limited the walks’ next moves to those that did not arrive in close vicinity of visited points. In other words, the grid-walking agent remembered a configured number of its previous locations and made sure that it did not approach them too closely. While implementing such behavior efficiently could pose challenges, we took a trial-and-error approach. In particular, the agent walked randomly and aborted its entire path to embark on a new attempt when a violation occurred. Since the kernels lengths we worked with were relatively short, we generated a sufficient number of kernels by setting the number of attempts to 256 for each kernel.

**Figure 2:**
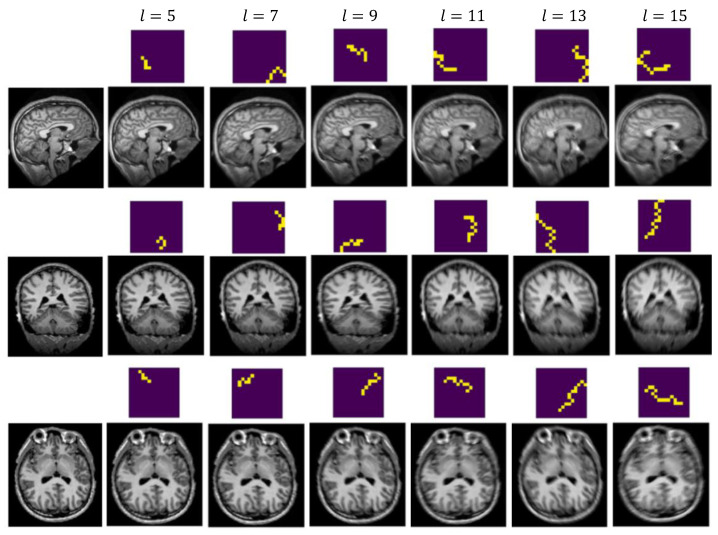
Sample Synthetic Images. Column 1 presents the original sharp images. Columns 2–7 demonstrate generated motion-affected images with kernel length = 5, 7, 9, 11, 13, 15, respectively. The respective convolutional kernel is shown on top of each synthetic image. Images better viewed when zoomed in.

Figure 2 presents sample synthetic blurred images generated using our random walk algorithm. Column 1 presents the original sharp images. Columns 2–7 demonstrate simulated motion-affected images with selective kernel lengths *l* = 5, 7, 9, 11, 13, and 15, respectively. The corresponding convolutional kernel is shown on top of each MR image. In our study, We generated kernels with all sizes up to a maximum length of 15. This limit was experimentally selected as we observed that longer settings led to over-blurred images that were unrealistic in simulating motion artifacts resulting from in-scanner head moves.

**Figure 3:**
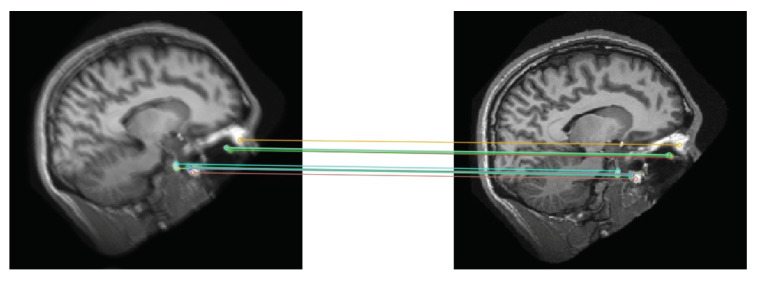
Alignment of Input and Target Images Using Matching Landmarks. (**Left**): image with synthetic blur after applying a random kernel. (**Right**): target image.

Because a 2D convolution may cause positional shifts from the original image, which may hinder its role as the ground-truth for the degraded (i.e., input) image. To address this issue, we resorted to OpenCV2’s ORB algorithm [41] to detect landmarks in a given image. The algorithm aligns two images by computing the landmark descriptors belonging to both images and matching the landmarks using a brute force algorithm (Figure 3). We further adjusted the intensity histogram of the aligned image to match that of the original image to prevent pixel intensity shifts.

## 5. Results

We trained our models on a PowerEdge R740 Linux machine with two Xeon 2.60 GHz CPUs (12 cores), 192 GB of memory, and a 32 GB NVIDIA Tesla V100 GPU. The training time is approximately 96 h for each model. Each model was trained using a 4:1 training and validation split on the synthetic datasets. We trained our models for 250 epochs with a batch size of 16. The training converged utilizing the Adam optimizer and a learning rate of 0.0001, minimizing the loss function defined in Equation (3).

### 5.1. Quantitative Evaluation Metrics

We evaluated the performance of our models using the following three quantitative metrics. The first two metrics were employed to assess the efficacy of the models for synthetic images in which the ground-truths (i.e., target images) are available. The third one was used to evaluate real-world images without ground truth. Furthermore, each output image was histogram-matched with the original image before these measures were calculated.

- Root Mean Square Error (RMSE): RMSE measures pixel-wise root mean square error between a pair of images. A smaller RMSE indicates a higher similarity between the images. We compare the RMSEs of the original blurred image and corrected model output against the ground-truth motion-free image.
- Peak Signal to Noise Ratio (PSNR) [42]: PSNR is the ratio between the maximum possible power of a signal and the power of corrupting noise that affects the fidelity of its representation. Thus, a higher PSNR indicates a higher quality of an image. We calculated the PSNRs after scaling pixel intensities of the images to the interval [0, 255].
- Perception-based Image Quality Evaluator (PIQE) [43]: PIQE evaluates the image quality using two psychovisually-based fidelity estimates: block-wise distortion and similarity. The two estimates are combined into a single PIQE score to assess quality. The smaller the value of PIQE, the better the image quality.

### 5.2. Model Performance

#### Evaluation on Synthetic Images

Table 1 presents the quantitative evaluation of our MC-GAN models on a synthetic dataset with ground truth. We further examined our MC-GAN model with data from individual anatomical planes, conjecturing that the structural similarity of the data could potentially mitigate the challenge and lead to improved model performance. The three directional models, denoted as MC-GAN(x), MC-GAN(y), and MC-GAN(z), represent models trained with data exclusively from the sagittal, axial, and coronal planes, respectively. The MC-GAN(xyz) denotes the model trained with images from all three directions. We further examine the breakdown performance of MC-GAN(xyz) along each anatomical plane indicated by the x-, y-, and z- directions in each MC-GAN(xyz) block.

**Figure 4:**
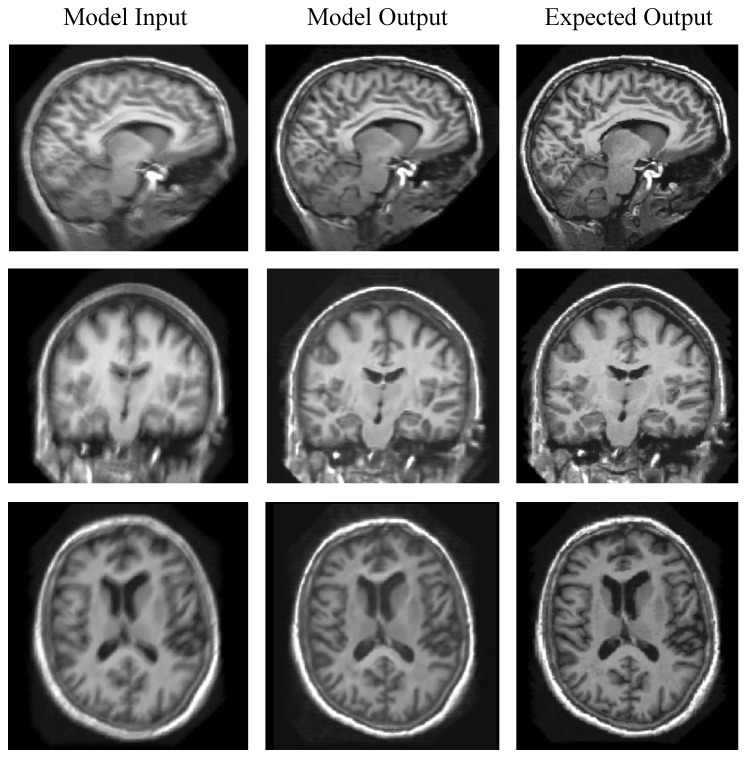
Visual Assessment of MC-GAN on Reducing Synthetic Motion Blurs. Left column: simulated motion blurs using random kernels (Section 4.2.2). Middle column: model-corrected output. Right column: ground truth images. From top to bottom rows are images from sagittal, coronal, and axial planes, respectively.

We evaluated our models’ performance across a spectrum of five degradation levels (Column 1), each of which contained 1000 motion-affected images whose PSNR scores with respect to the ground truth were within the indicated intervals. The “Degraded vs. Target” columns present the discrepancies (RMSE) and similarities (PSNR) between the blurred scans and their artifact-free counterparts in each category. The “Corrected vs. Target” columns present the discrepancies/similarities between the model-corrected images and the targets. The numbers in parentheses are standard deviations. The “Reduction” and “Gain” columns calculate the improvements of the model-corrected over the original images measured against the ground truth in RMSE and PSNR, respectively. Figure 4 demonstrates the efficacy of MC-GAN on a sample of synthetic test images.

We observe that the improvement made by the models decreases as the PSNR category (column 1) increases. This is an expected outcome because a higher PSNR indicates closer quality to the ground truth and, thus, less degradation. We view this as a desirable feature in that the models refrained from making substantial changes when the input image quality was high and intervened aggressively for those images with severe motion blurs.

Additionally, depending on the degradation level, MC-GAN(xyz) model achieved a 25.12% to 32.65% reduction in RMSE and a 2.62 to 3.76 dB gain in PSNR on a 5000-sample set of synthetic test images. Diving further into the model’s performance along each anatomical plane, we observe that MC-GAN(x) outperforms the x-direction of MC-GAN(xyz) in both RMSE and PSNR across all degradation categories. This outcome is consistent with our conjecture, which suggests MC-GAN(x) is more desirable over MC-GAN(xyz) for sagittal images. The same is true for the y- and z- directions in which MC-GAN(y) and MC-GAN(z) outperformed MC-GAN(xyz) in the respective directions across all degradation categories. Thus, we believe that it is more desirable to employ individual models trained using exclusive images from each anatomical plane.

Figure 4 illustrates MC-GAN’s action on motion blur in sample synthetic test images. The left column consists of degraded images (i.e., input to the models). The model’s output is shown in the middle column. The right column displays the expected output (i.e., ground truth images). Qualitatively, when presented with synthetically generated artifacts, the model appears to be highly effective.

### 5.3. Evaluation on Real-World Scans

Because real-world scans have no ground-truth images, we resorted to the perception-based image quality evaluator (PIQE) [43]. Table 2 presents the average PIQE scores for each model over 16,500 real-world motion-affected images described in Section 4. The results suggest that the outcome of the individual MC-GAN models (first three rows) exhibit notable improvements compared to the original images. The gain is particularly salient in the *z* direction with a 44.88% reduction in average PIQE scores. We also observe that MC-GAN(xyz) is less effective than the individual models, which is consistent with our findings in the synthetic data.

**Figure 5:**
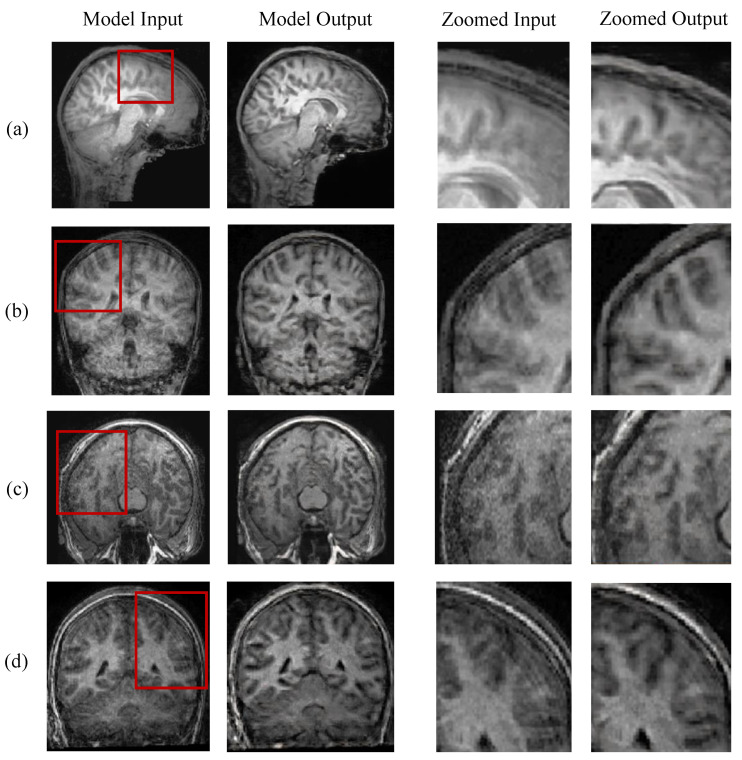
Visual Assessment of MC-GAN on Real-world Motion-affected Images. Images under the “Model Input” columns are original MR images; model-corrected output is displayed to the right. The two columns on the right show the zoomed-in regions indicated by the red boxes. Samples are selected from the sagittal (**a**), coronal (**b**,**d**), and axial (**c**) directions.

Figure 5 presents the qualitative assessment of MC-GAN on a sample of real-world images with motion artifacts. Images under the “Model Input” columns are original MR images, and the model-corrected outcomes are displayed to the right of each image. Although the improvements realized by the model on real-world scans are not as pronounced as those achieved on the synthetic images, we are encouraged by some observations. First, there is some transfer of the model’s strong performance in the synthetic domain to the real one. Specifically, our model made a positive enhancement to the original corrupted image in each case presented in Figure 5. Second, although the blind deconvolution problem is discouragingly difficult due to its intractability, our experiments indicate that deep neural networks can produce quality gains in real images by learning a collection of corrupting kernels using synthetic images. Thus, enhancing our kernels to simulate more comprehensive subject motions during MRI could lead to greater quality gains. Lastly, we observe that in Figure 5c, the output image may not be as sharp as the input image, but has less pixelated noise than the original image. The models further reduced the “ringing” artifacts in Figure 5b,d. We attribute these gains to the generative nature of GAN-based models. In particular, although our models were trained based on pairs of blurred and sharp images, they produced output images based on the learned distribution of the ground truth images and, thus, could remove additional artifacts to a limited degree.

## 6. Discussion

In this study, we proposed a technique to generate artificial motion blurs in brain MRI scans to address the data limitation issue in training deep learning models. Our method leveraged a collection of 10,000 random convolutional kernels designed to simulate in-scanner head motions. We further evaluated the efficacy of a GAN-based deep learn approach capitalizing on a large synthetic dataset generated using the proposed technique.

Our experimental results on synthetic and real-world MR images endorse our approach in that deconvolutions based on a large family of random kernels improved the degraded images’ quality with quantitative and qualitative evaluations. Nevertheless, we recognize that a more efficient process of densely populating the kernel space could illuminate the nature of typical corruptions and produce more robust real-life results. In particular, we assume that our constrained random walk process uniformly covers the kernel space. Although we have not validated this assumption theoretically, we believe that our relatively large kernel set achieved a sufficient coverage to produce perceptible results in practice. A potential enhancement could be to systematize the notion of kernel space coverage and examine the dependence of our method’s performance on the level of such coverage. Furthermore, the kernel generation process could be improved by introducing a relation of similarity between them (e.g., translation produces indistinguishable kernels) and producing kernel space coverings consisting of sufficiently dissimilar kernels.

It is worth noting that our study focused on reducing only motion blurs in degraded MR images. In practice, a corrupted MRI scan may contain a complex mixture of different types of motion artifacts, including rings, ghosting, signal dropouts, and unwanted signal enhancements. Thus, a potential future direction for this research could be to develop a more comprehensive process to simulate the heterogeneous nature of real-world motion artifacts.

Lastly, our experimental results suggest merit in training individual models for the respective anatomical planes. One explanation is that the input images’ structural similarity narrowed the problem domain and contributed to the success of these customized models. We believe this area has not been extensively studied due to the limited availability of real-world labeled data. Our study fills this gap using data augmentation techniques that simulate both sharp and degraded MRI scans. One limitation of our directional models is that they are trained for the three orthogonal planes (i.e., sagittal, coronal, and axial). In practice, MRI scans can be performed along oblique planes. We expect similar approaches can be used to build effective models for other scan directions with corresponding sample images. Our approach can also be applied to other medical imaging modalities, including X-ray, B-scans, and computed tomography (CT) scans.

## 7. Conclusions

In this study, we investigated the viability of a new blind deconvolution approach to address motion blurs in brain MRI scans. In particular, a conditional adversarial network was trained to guess the deblurring kernel of a corrupted image based on a relatively small universe of “basis” kernels. To facilitate training effective deep learning models, we applied data augmentation techniques and generated a large number of realistic synthetic brain MRI images. Our experimental results suggested that, with a sufficiently dense basis, a neural network could effectively guess the degrading kernel and improve the image quality of motion-affected scans. Our study further demonstrated values in building customized models for individual MRI planes. We recognize some limitations associated with our framework, including the primitive approximation of complex real-world head motions and the assumption of a uniform kernel space coverage with our random walk approach. Nevertheless, we believe that the framework presented here merits further investigation based on the promise shown by our experiments. 

## Figures and Tables

**Table 1:** Quantitative Evaluation on Synthetic Data Across Different Degradation Levels.

PSNR Level	Model	Pixel-Wise RMSE			PSNR (dB)		
		Degraded vs. Target	Corrected vs. Target	Reduction (%)	Degraded vs. Target	Corrected vs. Target	Gain
<17	MC-GAN (x)	0.162 (0.022)	0.115 (0.034)	**29.45%**	15.85 (1.04)	19.18 (2.50)	**3.33**
	MC-GAN (y)	0.161 (0.025)	0.097 (0.035)	**40.03%**	15.93 (1.06)	20.82 (2.95)	**4.89**
	MC-GAN (z)	0.167 (0.028)	0.101 (0.045)	**39.45%**	15.66 (1.22)	20.60 (3.30)	**4.94**
	MC-GAN (xyz)	0.163 (0.024)	0.110 (0.035)	**32.65%**	15.81 (1.10)	19.56 (2.59)	**3.76**
	x-direction	0.162 (0.022)	0.120 (0.032)	26.43%	15.85 (1.04)	18.75 (2.26)	2.90
	y-direction	0.161 (0.025)	0.097 (0.031)	39.58%	15.93 (1.06)	20.61 (2.47)	4.67
	z-direction	0.167 (0.028)	0.102 (0.039)	38.97%	15.66 (1.22)	20.33 (2.73)	4.67
[17, 18)	MC-GAN (x)	0.133 (0.004)	0.097 (0.023)	**27.31%**	17.53 (0.27)	20.53 (2.02)	**3.00**
	MC-GAN (y)	0.132 (0.005)	0.086 (0.025)	**35.37%**	17.57 (0.30)	21.72 (2.50)	**4.15**
	MC-GAN (z)	0.132 (0.004)	0.090 (0.028)	**30.79%**	17.56 (0.29)	21.17 (2.68)	**3.60**
	MC-GAN (xyz)	0.133 (0.004)	0.095 (0.021)	**28.39%**	17.55 (0.28)	20.67 (1.96)	**3.12**
	x-direction	0.133 (0.004)	0.100 (0.021)	24.45%	17.53 (0.27)	20.14 (1.74)	2.61
	y-direction	0.132 (0.005)	0.089 (0.021)	32.37%	17.57 (0.30)	21.20 (2.01)	3.62
	z-direction	0.132 (0.004)	0.090 (0.022)	30.74%	17.56 (0.29)	21.12 (2.05)	3.55
[18, 19)	MC-GAN (x)	0.120 (0.004)	0.085 (0.019)	**28.50%**	18.45 (0.28)	21.57 (1.89)	**3.12**
	MC-GAN (y)	0.118 (0.004)	0.08 (0.022)	**32.81%**	18.54 (0.28)	22.3 (2.34)	**3.77**
	MC-GAN (z)	0.119 (0.004)	0.079 (0.023)	**33.15%**	18.52 (0.28)	22.36 (2.45)	**3.84**
	MC-GAN (xyz)	0.119 (0.004)	0.085 (0.019)	**28.34%**	18.50 (0.29)	21.60 (1.92)	**3.10**
	x-direction	0.120 (0.004)	0.090 (0.017)	24.77%	18.45 (0.28)	21.07 (1.62)	2.62
	y-direction	0.118 (0.004)	0.084 (0.019)	29.04%	18.54 (0.28)	21.73 (1.96)	3.20
	z-direction	0.119 (0.004)	0.082 (0.020)	31.30%	18.52 (0.29)	22.02 (2.04)	3.50
[19, 20)	MC-GAN (x)	0.107 (0.003)	0.077 (0.016)	**27.97%**	19.43 (0.28)	22.45 (1.71)	**3.02**
	MC-GAN (y)	0.106 (0.004)	0.071 (0.018)	**33.31%**	19.48 (0.29)	23.26 (2.14)	**3.79**
	MC-GAN (z)	0.106 (0.004)	0.071 (0.019)	**33.50%**	19.48 (0.29)	23.31 (2.26)	**3.84**
	MC-GAN (xyz)	0.106 (0.004)	0.076 (0.016)	**28.60%**	19.47 (0.29)	22.57 (1.76)	**3.10**
	x-direction	0.107 (0.003)	0.083 (0.015)	22.79%	19.43 (0.28)	21.80 (1.49)	2.37
	y-direction	0.106 (0.004)	0.075 (0.016)	29.66%	19.48 (0.29)	22.72 (1.80)	3.24
	z-direction	0.106 (0.004)	0.073 (0.015)	31.05%	19.48 (0.29)	22.88 (1.73)	3.40
>20	MC-GAN (x)	0.089 (0.009)	0.067 (0.012)	**24.49%**	21.09 (0.97)	23.61 (1.53)	**2.53**
	MC-GAN (y)	0.088 (0.010)	0.061 (0.014)	**30.59%**	21.19 (1.13)	24.50 (1.90)	**3.32**
	MC-GAN (z)	0.089 (0.010)	0.064 (0.015)	**27.60%**	21.11 (1.06)	24.06 (1.89)	**2.96**
	MC-GAN (xyz)	0.088 (0.010)	0.066 (0.013)	**25.12%**	21.14 (1.08)	23.76 (1.69)	**2.62**
	x-direction	0.089 (0.009)	0.071 (0.012)	20.44%	21.09 (0.97)	23.14 (1.42)	2.05
	y-direction	0.088 (0.010)	0.064 (0.013)	27.33%	21.19 (1.13)	24.06 (1.69)	2.87
	z-direction	0.089 (0.010)	0.067 (0.014)	24.22%	21.11 (1.06)	23.62 (1.71)	2.52

The “Degraded vs. Target” columns present the discrepancies (RMSE) and similarities (PSNR) between blurred scans and their artifact-free counterparts in each category. The “Corrected vs. Target” columns show the discrepancies/similarities between model-corrected images and the targets. The values were computed after first scaling the images to the range [0, 255]. The numbers in parentheses are standard deviations. Bold numbers show each model’s overall RMSE reduction and PSNR gain. The x-, y-, z- directions present the breakdown performance of MC-GAN(xyz) along the sagittal, axial, and coronal planes, respectively.

**Table 2:** Quantitative Evaluation on Real-world ABIDE data.

Models	PIQE		
	Degraded	Corrected	Reduction (%)
MC-GAN (x)	9.09 (3.77)	7.98 (5.23)	**12.26%**
MC-GAN (y)	12.17 (6.62)	9.01 (7.52)	**26.01%**
MC-GAN (z)	12.45 (10.65)	6.86 (5.05)	**44.88%**
MC-GAN (xyz)	11.24 (7.71)	9.11 (7.00)	**18.97%**
x-direction	9.09 (3.77)	8.38 (5.65)	7.84%
y-direction	12.17 (6.62)	9.75 (7.13)	19.92%
z-direction	12.45 (10.65)	9.19 (7.14)	26.18%

The “Degraded” column measures the PIQE between blurred scans and their artifact-free counterparts. The “Corrected” column measures the PIQE between model-corrected images and the targets. The x-, y-, z- directions present the breakdown performance of MC-GAN(xyz) along the sagittal, axial, and coronal planes, respectively. The numbers in parentheses are standard deviations. Bold numbers indicate each model’s PIQE reduction. The x-, y-, z- directions present the breakdown performance of MC-GAN(xyz) along the sagittal, axial, and coronal planes, respectively.

## Data Availability

Data for this research can be accessed from the open access platforms provided in references [17,18].
